# A novel C-terminal DxRSDxE motif in ceramide synthases involved in dimer formation

**DOI:** 10.1016/j.jbc.2021.101517

**Published:** 2021-12-20

**Authors:** Jiyoon L. Kim, Shifra Ben-Dor, Eden Rosenfeld-Gur, Anthony H. Futerman

**Affiliations:** 1 Department of Biomolecular Sciences, Weizmann Institute of Science, Rehovot, Israel; 2 Life Sciences Core Facilities, Weizmann Institute of Science, Rehovot, Israel

**Keywords:** ceramide, ceramide synthase, dimer, motif, sphingolipids, CerS, ceramide synthase, HA, hemagglutinin, Hek, human embryonic kidney, LC, long-chain, NBD-Sph, NBD-sphinganine, SL, sphingolipid, TLC, Tram-Lag-CLN8, UPLC, Ultra Performance Liquid Chromatography, VLC, very-long-chain

## Abstract

Ceramide is a lipid moiety synthesized *via* the enzymatic activity of ceramide synthases (CerSs), six of which have been identified in mammalian cells, and each of which uses a unique subset of acyl-CoAs for ceramide synthesis. The CerSs are part of a larger gene family, the Tram-Lag-CLN8 domain family. Here, we identify a unique, C-terminal motif, the DxRSDxE motif, which is only found in CerSs and not in other Tram-Lag-CLN8 family members. Deletion of this motif in either CerS2 or in CerS6 did not affect the ability of either enzyme to generate ceramide using both an *in vitro* assay and metabolic labeling, but deletion of this motif did affect the activity of CerS2 when coexpressed with CerS6. Surprisingly, transfection of cells with either CerS2 or CerS6 lacking the motif did not result in changes in cellular ceramide levels. We found that CerS2 and CerS6 interact with each other, as shown by immunoprecipitation, but deletion of the DxRSDxE motif impeded this interaction. Moreover, proteomics analysis of cells transfected with CerS6^Δ338–344^ indicated that deletion of the C-terminal motif impacted cellular protein expression, and in particular, the levels of ORMDL1, a negative regulator of sphingolipid synthesis. We suggest that this novel C-terminal motif regulates CerS dimer formation and thereby impacts ceramide synthesis.

Levels of cellular sphingolipids (SL) depend on a delicate balance between biosynthetic and degradative pathways (1). Central to both of these pathways are the ceramide synthases (CerSs) (2), which are able to *N*-acylate sphinganine (d18:0) generated during biosynthesis and also *N*-acylate sphingosine (d18:1) (3), which is generated *via* the recycling pathway of SL degradation (4). The CerSs are subject to a number of mechanisms that regulate their activity, which include phosphorylation (5) and dimerization (6), among the known post-translation modes of regulation.

The defining feature of the mammalian CerSs is that each of the six isoforms uses a distinct set of acyl CoAs for (dihydro)ceramide synthesis so as to generate ceramides (7) and up-stream complex SLs such as sphingomyelin and glycosphingolipids, with defined *N*-acyl chain lengths. Of relevance to the present study is the use of C22-C24-acyl CoAs by CerS2 (8) and C16-CoAs by CerS6 (9). Some progress has been made in identifying how these homologous enzymes generate ceramide acyl chain length specificity (10), with a region of 11 residues in a putative cytosolic loop recently identified as playing a critical role in determining CerS specificity, at least of CerS2 and CerS5/6 (11).

Although the *N*-acyl CoA specificity of CerSs is now well-established (10, 11, 12), an intriguing observation from some years ago has not been fully investigated. Expression of two CerSs together demonstrated that the activity of one CerS affects the activity of the other, without altering their specificity, *via* the formation of CerS homo and heterodimers (6). For instance, in a constitutive heterodimer comprising CerS5 and CerS2, the activity of CerS2 depends on a catalytically active CerS5, leading to the suggestion that ceramide synthesis could be regulated by the formation of CerS dimers. This implies that the *N*-acyl composition of SLs in cells not only depends on the expression pattern of CerSs in any one particular cell type (8) but also depends upon dimer formation between pairs of CerSs.

While further pursuing the mode of interaction of CerS dimers, we now identify a unique, C-terminal motif, the DxRSDxE motif (amino acids 338–344 in CerS6 and in CerS2) which is only found in CerS and not found in other members of the protein family to which the CerSs belong, namely the Tram-Lag-CLN8 (TLC) domain family (13). Deletion of this motif in CerS6 does not affect the ability of CerS6 to synthesize C16-ceramide *in vitro*, but unexpectedly, the levels of cellular ceramides are unaltered upon transfection with CerS6^Δ338–344^. However, this motif affects the ability of CerS to form homo and heterodimers. Together, our studies have identified a novel motif which adds to the complexity of understanding how the CerSs are regulated and thus how SL levels in cells are determined.

## Results

### The DxRSDxE motif differentiates between CerSs and other TLC family members

CerSs, known at the time as longevity assurance gene homologs (14) were originally characterized as members of the TLC domain-containing family of proteins (13, 15). The CerSs contain a number of functional domains, including a Hox-like domain (16) (with the exception of mammalian CerS1), the TLC domain itself, and a C-terminus region of differing lengths, with little homology identified between the various CerSs in the C-terminus (2). A number of splice variants have also been identified at the C-terminus of CerS1, CerS5, and CerS6 (7, 15, 17, 18, 19).

**Figure 1:**
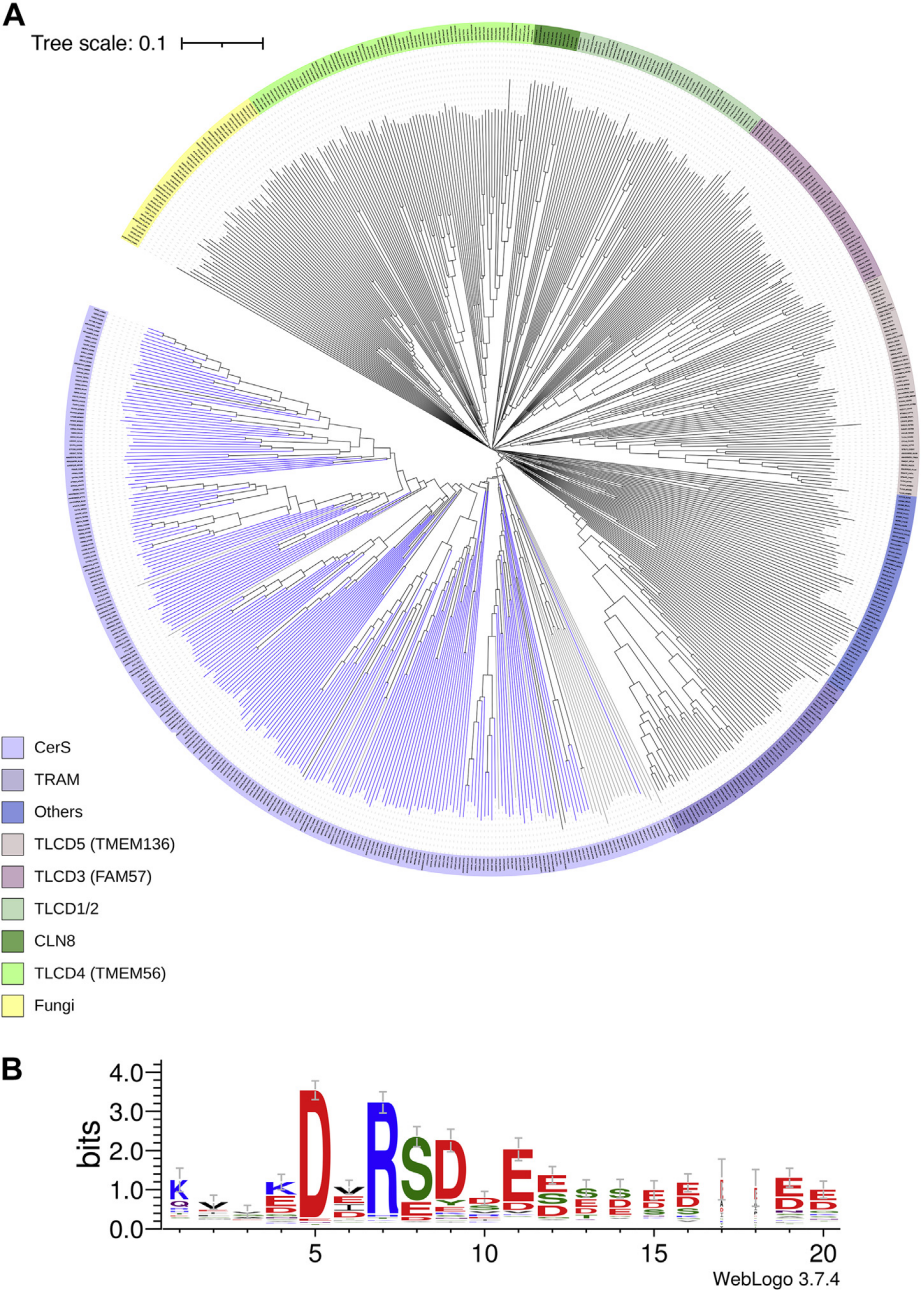
**A novel motif in the C-terminus of CerS.***A*, phylogenetic tree of proteins with a TLC domain. Full length protein sequences were taken from 90 species for a total of 630 TLC domain-containing proteins. The tree clearly distinguishes between clades, named according to the human proteins when possible. *Blue*, DxRSDxE-containing CerS; *gray*, proteins with an incomplete C-terminal sequence; and *black*, TLC proteins which do not contain the DxRSDxE motif. *B*, the DxRSDxE motif from the CerS branch of the tree (209 sequences from 84 species) is located in the initial part of the C-terminus. For further details, see Table S1. CerS, ceramide synthase; TLC, Tram-Lag-CLN8.

**Figure 2:**
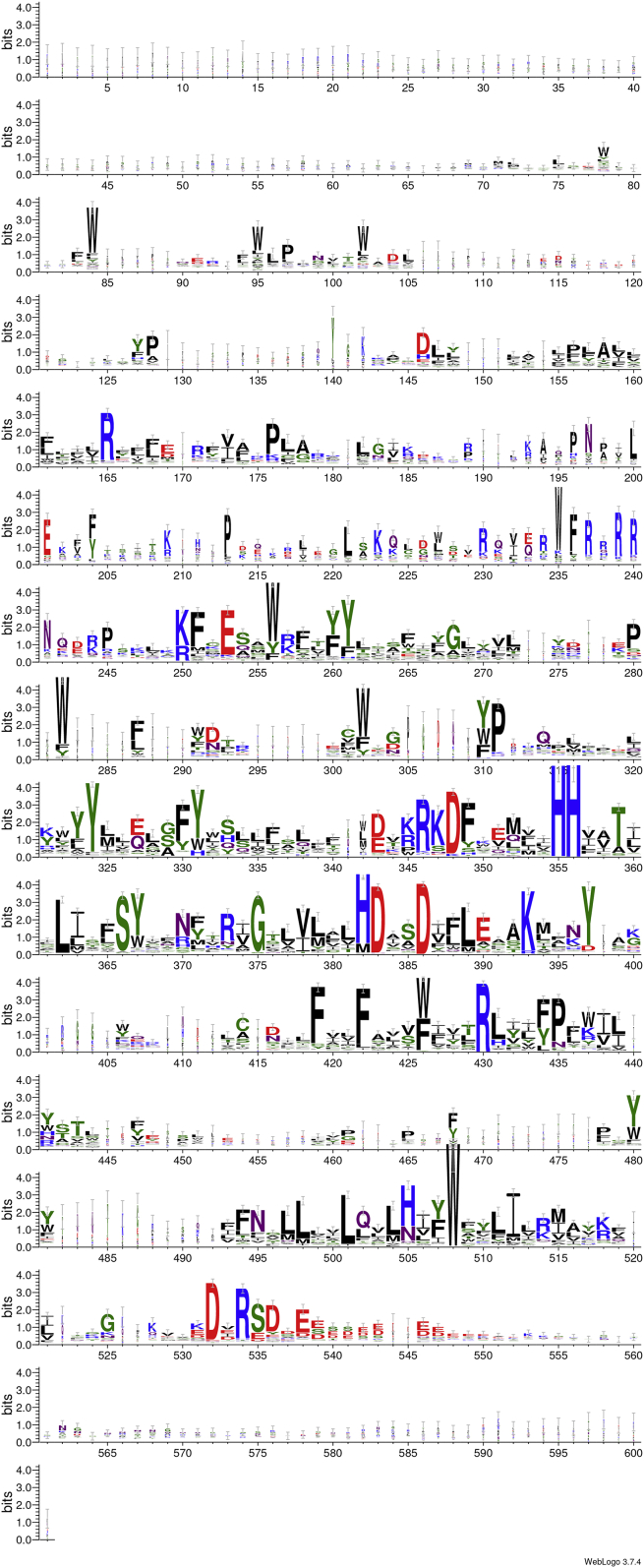
**The full length logo of the CerS clade.** A full length view of the logo of the CerS clade (209 sequences from 84 species), where the Lag1p motif (13, 24, 26), the putative active site, can be seen in lines 9 to 10, and the DxRSDxE motif (highlighted in Fig. 1*B*) in line 14. The alignment was pruned to remove columns with less than ten sequences before logo generation. CerS, ceramide synthase; HA, hemagglutinin.

To further study the phylogenetic relationships between members of the TLC domain family and to determine if additional domains and motifs can be identified, phylogenetic trees were constructed using TLC proteins from 90 organisms over a wide phylogenetic range (Fig. 1*A*, see additional details in Table S1). The tree splits into clearly defined clades, named here according to the human proteins when possible, and the major species if not. The CerSs are the major clade, comprising approximately one third of the tree. The other clades either have poorly defined function or no known function. The TRAM proteins, which are involved in transport of proteins across the ER membrane (20), are the next closest clade to the CerSs. Two other clades of the family, FAM57B and CLN8, appear to be involved in the regulation of ceramide levels, although they do not synthesize ceramide directly (21, 22). The active sites of the various proteins have likewise not been unambiguously identified although a double histidine motif (amino acids 212–213 in CerS2 and 211–212 in CerS6) has been suggested to be the active site, although it is also present in other TLC domain-containing proteins, with the exception of TRAM and the fungi clades. Upon alignment of the full length protein sequences (Fig. 2), a short motif was found exclusively in the CerS clade (blue branches in Fig. 1*A*), with the sequence DxRSD followed by either D or E (designated DxRSDxE herein, Fig. 1*B*). This motif is located at the C-terminal end of the TLC domain and although it is not normally considered part of the TLC domain, its conservation in the CerS clade might suggest that, at least in the CerSs, it should be considered a *bone fide* part of the TLC domain.

### Deletion of the DxRSDxE motif affects ceramide synthesis upon coexpression of more than one CerS

**Figure 3:**
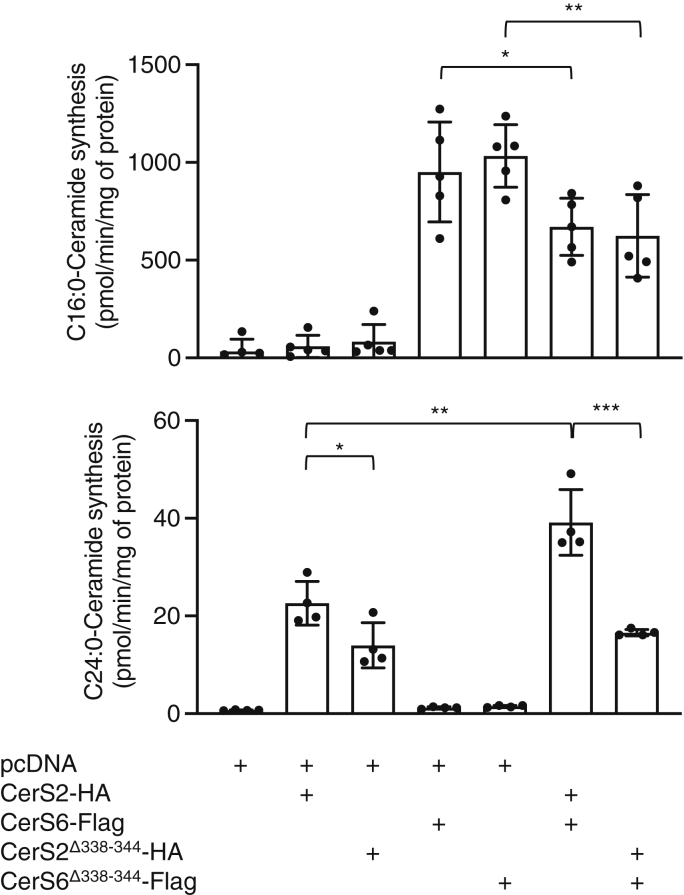
**The effect of deletion of the DxRSDxE motif on CerS activity *in vitro*.** CerS2^−/−^ Hek 293T cells were transfected with the indicated CerS and CerS activity assayed using C16:0- (*upper panel*) or C24:0-CoA (*lower panel*). The data are means ± S.D., n = 4 to 5. ∗*p* < 0.05; ∗∗*p* < 0.01; ∗∗∗*p* < 0.001. CerS, ceramide synthase; Hek, human embryonic kidney.

**Figure 4:**
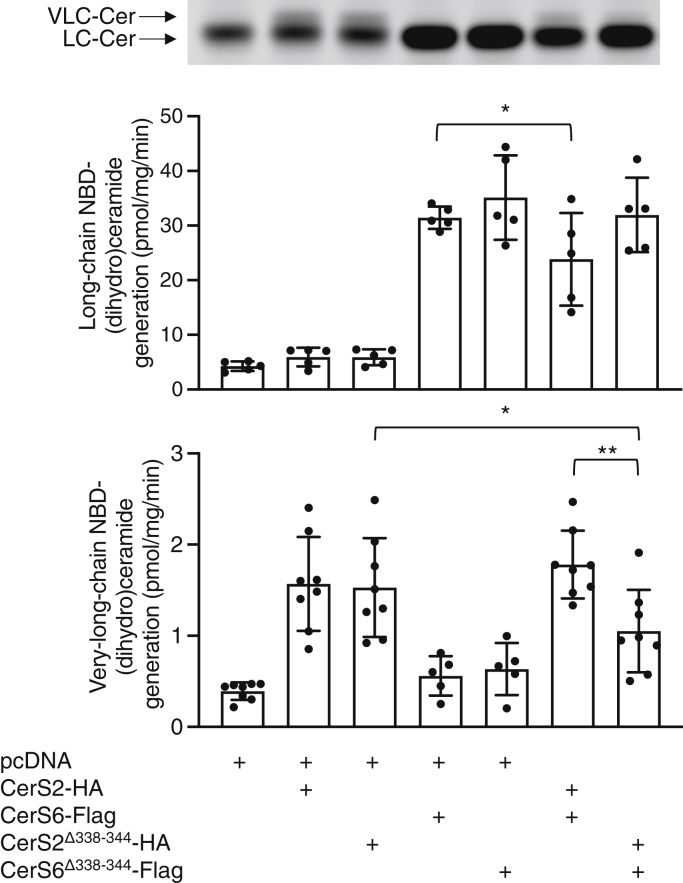
**The effect of deletion of the DxRSDxE motif on CerS activity measured by metabolic labeling with NBD-Sph.** CerS2^−/−^ Hek 293T cells were transfected with the indicated CerS for 48 h. The cells were then incubated with NBD-Sph for 10 min before lipid extraction. VLC- and LC-NBD-(dihydro)ceramides were separated by thin layer chromatography (an example of the separation is shown in the *upper panel*). The data are means ± S.D., n = 4. n.s., not significant; ∗*p* < 0.05; ∗∗*p* < 0.01. CerS, ceramide synthase; HA, hemagglutinin; Hek, human embryonic kidney; LC, long-chain; NBD-Sph, NBD-sphinganine; VLC, very-long-chain.

Because the DxRSDxE motif can be considered part of the TLC domain in CerSs, we first examined whether it is directly involved in modulating levels of ceramide synthesis. Hemagglutinin (HA)-tagged CerS2 and Flag-tagged CerS6, along with the respective deletion constructs of the DxRSDxE motif (CerS6^Δ338–344^-Flag and CerS2^Δ338–344^-HA), were expressed in CerS2^−/−^ human embryonic kidney (Hek)293T cells, which do not contain any endogenous CerS2. Deletion of the DxRSDxE motif in CerS6-Flag had no discernable effect on ceramide synthesis when assayed *in vitro* (Fig. 3), in as much as C16-ceramide levels were elevated to a similar extent upon transfection with CerS6^Δ338–344^-Flag as with CerS6-Flag. CerS2^Δ338–344^-HA had slightly reduced activity compared to CerS2-HA, retaining ∼60% activity. This was confirmed upon metabolic labeling of cells with NBD-Sphinganine (NBD-Sph) and analysis of the amount of formation of NBD-(dihydro)ceramides by thin layer chromatography; note that thin layer chromatography can only distinguish long-chain (LC)-(dihydro)ceramides (*i.e.*, C16-C20) and very-long-chain (VLC)-(dihydro)ceramides (*i.e.*, C22-C26). The levels of VLC-NBD-(dihydro)ceramides were similarly increased upon transfection with CerS2^Δ338–344^-HA or CerS2-HA, and the levels of LC-NBD-(dihydro)ceramides were similarly increased upon transfection with CerS6^Δ338–344^-Flag or CerS6-Flag (Fig. 4). This indicates that deletion of the DxRSDxE motif has no effect on the ability of either of these CerSs to generate their respective ceramides under optimal kinetic conditions *in vitro*.

Previous studies have shown that CerS6 can modulate the activity of CerS2 with respect to the amount of C24-ceramide that CerS2 is able to generate (6). This was confirmed in the present study, at least using an *in vitro* CerS assay, inasmuch as coexpression of CerS6-Flag along with CerS2-HA increased levels of C24-ceramide synthesis (Fig. 3). However, this increase in C24-ceramide synthesis was not observed upon coexpression of the deletion constructs, that is, CerS6^Δ338–344^-Flag together with CerS2^Δ338–344^-HA, either using an *in vitro* assay (Fig. 3) or by metabolic labeling with NBD-Sph (Fig. 4), implying that interactions between CerS2 and CerS6 might be mediated by the DxRSDxE motif, and that C24-ceramide synthesis by CerS2 is particularly dependent on this interaction. In contrast, optimal CerS6 activity appears less dependent on the DxRSDxE motif (Figs. 3 and 4). These results are consistent with earlier data indicating that maximal CerS2, but not CerS6 activity, requires interaction with another CerS, in this case, CerS6 (6).

### The DxRSDxE motif regulates the interaction of CerS with each other

**Figure 5:**
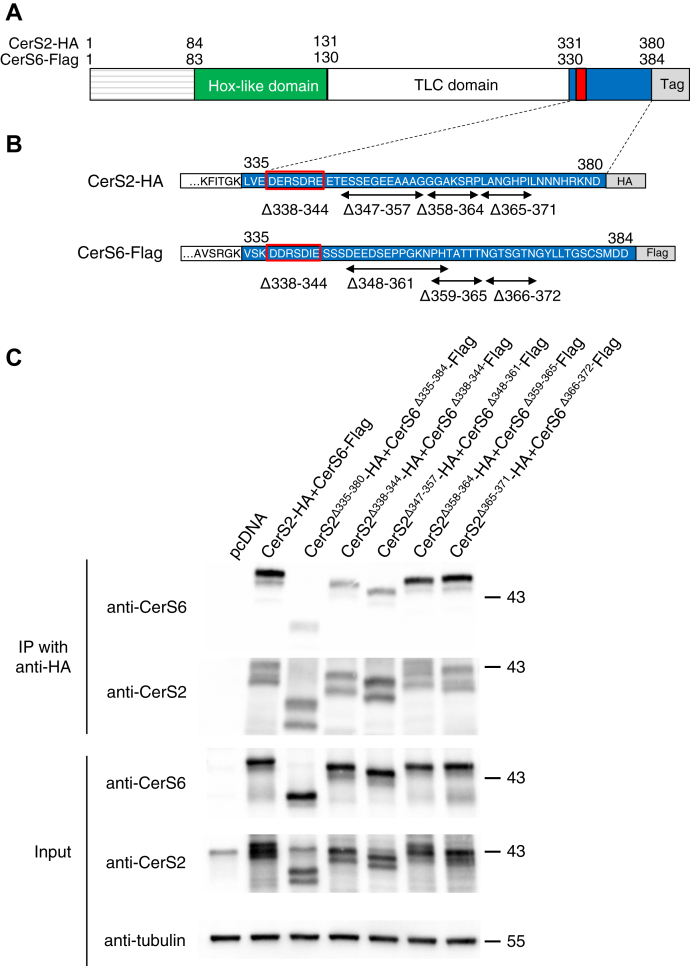
**The DxRSDxE motif regulates dimer formation between CerS2 and CerS6.***A*, schematic representation of the major CerS domains. Hox-like domain (*green*), TLC domain (*white*), C terminus (*blue*), DxRSDxE motif (*red*), and tags (*gray*) of human CerS2-HA and CerS6-Flag. *B*, an expanded view of the C-terminus indicating the various deletions that were generated and analyzed by coimmunoprecipitation. *C*, Hek293T cells were transfected with the indicated CerS or deletion constructs. 48 h after transfection, immunoprecipitation (IP) was performed using an anti-HA antibody (*upper panel*). Western blotting to determine the extent of coimmunoprecipitation was performed using either an anti-CerS6 or an anti-CerS2 antibody because the HA and Flag tags could not be detected when the C-terminus was deleted (Δ335–380 in CerS2 and Δ335–384 in CerS6) even though the tags were present (confirmed by sequencing). The levels of expression (*input*) after transfection are shown in the *lower panel*. The experiment was repeated three times with similar results. Mr are shown. CerS, ceramide synthase; HA, hemagglutinin; Hek, human embryonic kidney; TLC, Tram-Lag-CLN8.

We next directly tested whether deleting the DxRSDxE motif indeed affects the interaction of CerS6 with CerS2. CerS2-HA coimmunoprecipitated with CerS6-Flag (Fig. 5), but less coimmunoprecipitation was detected when the whole C-terminal region of both CerS were deleted (Fig. 5). Moreover, much less interaction was detected upon deletion of the DxRSDxE motif when CerS2^Δ338–344^-HA was expressed with CerS6^Δ338–344^-Flag. Deletion of another sequence of 11 (CerS2^Δ347–357^-HA) or 14 (CerS6^Δ348–361^-Flag) residues adjacent to the DxRSDxE motif (Fig. 5) also affected the extent of coimmunoprecipitation between CerS2-HA and CerS6-Flag, but deletion of two other sequences (CerS2^Δ358–364^-HA/CerS6^Δ359–365^-Flag or CerS2^Δ365–371^-HA/CerS6^Δ366–372^-Flag) further downstream to the DxRSDxE motif had no effect of the extent of coimmunoprecipitation. We conclude that the DxRSDxE motif is involved in or required for the interaction between CerS2 and CerS6. The deletion of a relatively long peptide directly adjacent to this motif impinges upon the ability of the motif to mediate this interaction, perhaps *via* a conformational or structural effect, whereas deletion of two other sequences further down-stream had no effect. Note that none of the other three sequences tested show any homology between the CerSs (Fig. 2).

### Deletion of the DxRSDxE motif does not affect lipid levels in cultured cells but does affect the pattern of protein expression

**Figure 6:**
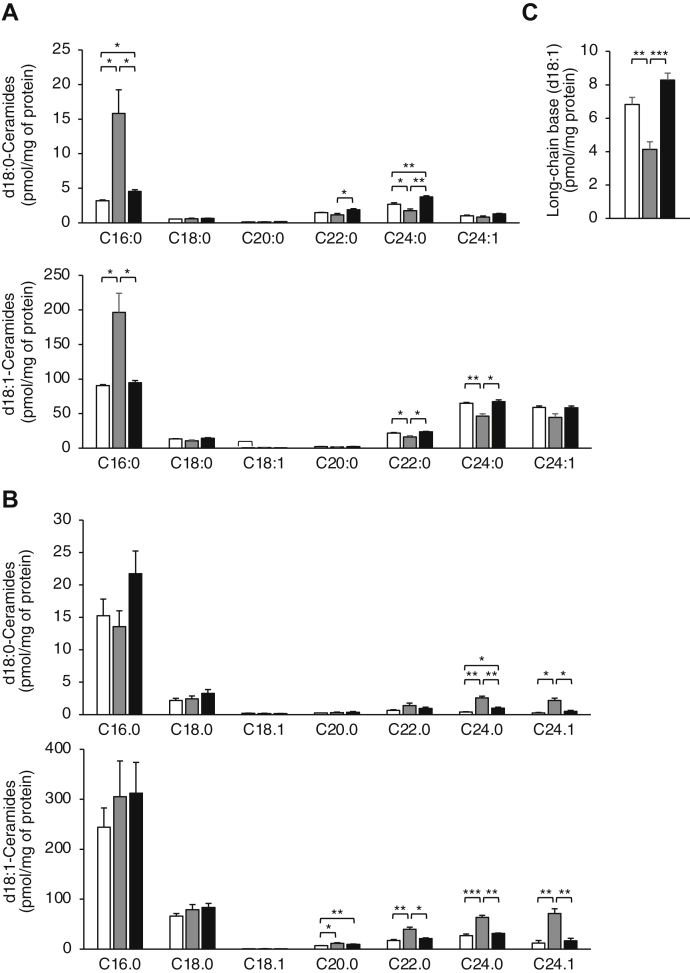
**The effect of deleting the DxRSDxE motif on cellular ceramide levels.***A*, fatty acyl composition of d18:0- and d18:1-ceramides measured by LC-MS/MS 48 after transfection with pcDNA (*white*), CerS6-Flag (*gray*), or CerS6^Δ338–344^-Flag (*black*) in Hek293T cells. *B*, fatty acyl composition of d18:0- and d18:1-ceramides 48 after transfection with pcDNA (*white*), CerS2-HA (*gray*), or CerS2^Δ338–344^-HA (*black*) in CerS2^−/−^ Hek293T cells. *C*, levels of d18:1-long-chain bases. The data are means ± S.E.M., n = 3 to 4. ∗*p* < 0.05; ∗∗*p* < 0.01; ∗∗∗*p* < 0.001. CerS, ceramide synthase; HA, hemagglutinin; Hek, human embryonic kidney.

To determine whether deletion of the DxRSDxE motif affects SL levels in cultured cells, the cells were transfected with either CerS6-Flag or with CerS6^Δ338–344^-Flag for 48 h before analysis by LC-MS/MS. The levels of C16:0-ceramide and C16:0-dihydroceramide (Fig. 6*A*) were significantly up-regulated upon transfection with CerS6-Flag, with a concomitant decrease in very-long chain ceramide levels, as expected based on the acyl chain specificity of CerS6 (9). Likewise, the levels of sphingosine decreased upon transfection with CerS6-Flag (Fig. 6*C*). Although there was a 40% increase in C16:0-dihydroceramide levels upon CerS6^Δ338–344^-Flag over-expression, in contrast, and unexpectedly in light of the ability of CerS6^Δ338–344^-Flag to synthesize ceramide *in vitro*, no changes in levels of C16:0-ceramide (Fig. 6*A*), or sphingosine (Fig. 6*C*) were detected upon transfection with CerS6^Δ338–344^-Flag; similar results were obtained upon analysis of C16:0-hexosylceramide and of C16:0-dihydrohexosylceramide levels, inasmuch as levels were increased upon transfection with CerS6-Flag but not with CerS6^Δ338–344^-Flag (data not shown). Upon transection with CerS2-HA in CerS2^−/−^ Hek293T cells, the levels of d18:0/C24:0 and d18:0/C24:1-ceramides were elevated (Fig. 6*B*), consistent with the known acyl chain specificity of CerS2 (8), but there were no changes in d18:0/C24:1-ceramide levels upon transfection with CerS2^Δ338–344^-HA (Fig. 6*B*).

Despite the lack of change in ceramide levels in cells transfected with either CerS2^Δ338–344^-HA or CerS6^Δ338–344^-Flag, we also analyzed ceramide levels in cells expressing both together. Ceramide and dihydroceramide levels were similar in cells expressing both Cer2-HA and CerS6-Flag, compared to cells expressing CerS2^Δ338–344^-HA and CerS6^Δ338–344^-Flag. Likewise, the cells had similar levels of C24:0- and C24:1-ceramide in cells expressing CerS2^Δ338–344^-HA and CerS6^Δ338–344^-Flag compared to cells expressing CerS2^Δ338–344^-HA alone (data not shown), consistent with the lack of change in ceramide levels in the cultured cells upon expression of each deletion construct individually.

**Figure 7:**
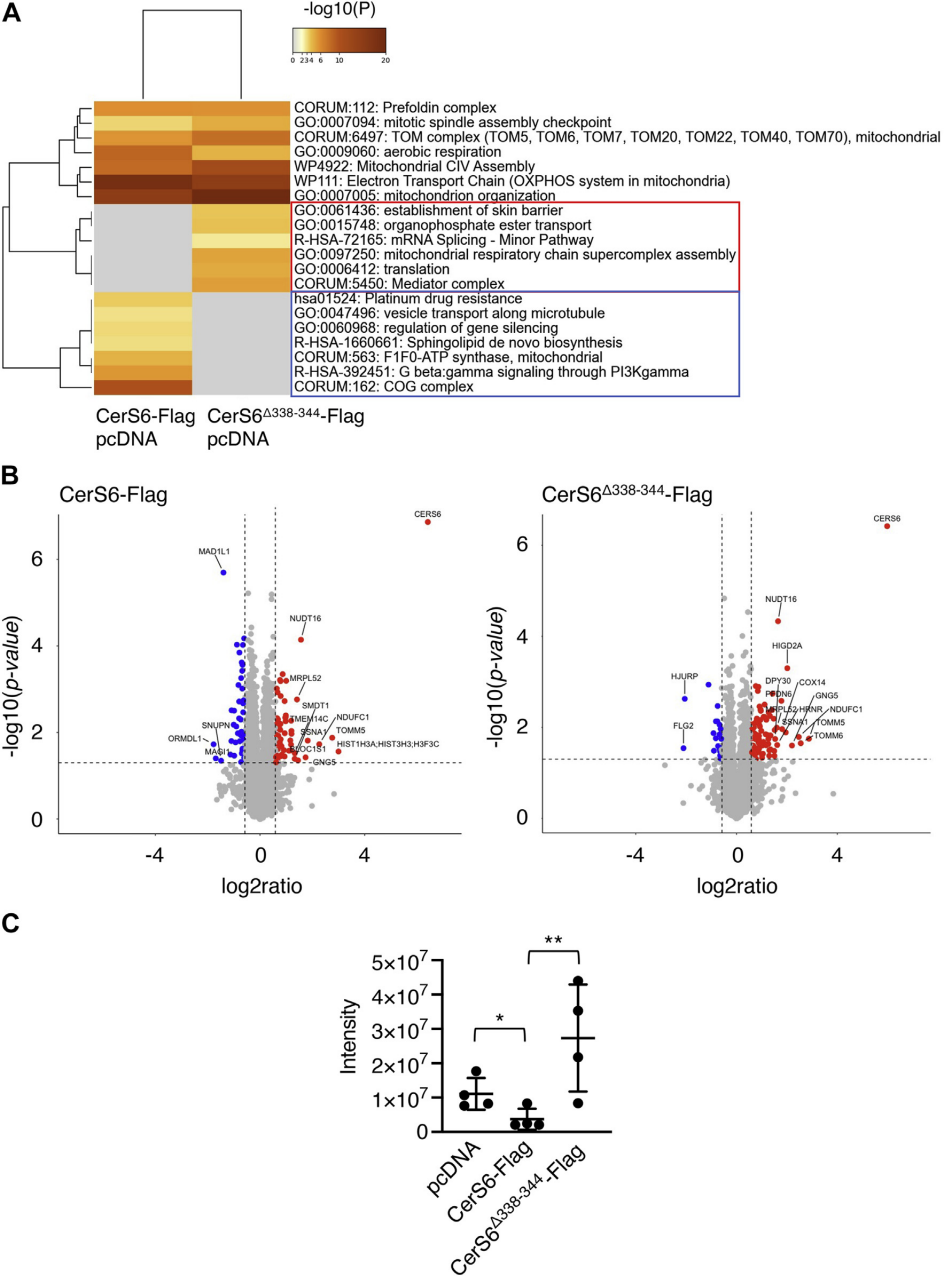
**Differentially expressed proteins upon expression of CerS6 and CerS6**^**Δ338–344**^**.** Hek293T cells were transfected with the indicated CerSs for 48 h before proteomics analysis. *A*, metascape heatmap of Gene Ontology (GO) and pathway-enriched terms colored by *p* values for the top nonredundant enrichment clusters. The enriched clusters altered in CerS6^Δ338–344^-Flag (*red*) or CerS6-Flag-transfected cells (*blue*) *versus* pcDNA. *B*, Volcano plots of differentially expressed proteins in CerS6-Flag or CerS6^Δ338–344^-Flag transfected cells compared to pcDNA-transfected cells. X = Log2ratio *versus* pcDNA, Y = −log10 (*p* value). The top 15 hits in each plot are shown in Tables 1 and 2. Differentially expressed proteins are shown in Table S3. The *dashed lines* are set at a *p* value of 0.05 (*y* axis) and ratio of 0.67 and 1.5 (*x* axis). The top 15 hits for up- (*red*) and down-regulated (*blue*) proteins are shown. *C*, scatter plot of ORMDL1 levels. The ORMDL1 protein can be identified by one unique peptide and four peptides used for measurements. The data are means ± S.D., n = 4. ∗*p* < 0.05; ∗∗*p* < 0.01; ∗∗∗*p* < 0.001. CerS, ceramide synthase; Hek, human embryonic kidney.

To determine whether deletion of the DxRSDxE motif nevertheless caused any changes in cell physiology, we performed nontargeted label-free proteomics of Hek 293T cells transfected with CerS6-Flag compared to cells transfected with CerS6^Δ338–344^-Flag. All identified proteins are documented in Table S2. Analysis of differentially expressed proteins (CerS6-Flag *versus* pcDNA, compared to CerS6^Δ338–344^-Flag *versus* pcDNA) by Metascape (https://metascape.org/) indicated that over-expression of CerS6-Flag leads to changes in pathways of vesicular transport, *de novo* SL synthesis, and the conserved oligomeric Golgi complex (Fig. 7*A*), whereas over-expression of CerS6^Δ338–344^-Flag resulted in changes in a different set of pathways, which included translation and the mitochondria respiratory pathway (Fig. 7*A*). A number of pathways were common to both, including some aspects of mitochondria biology (*i.e.*, electron transport chain, complex IV assembly). More detailed analysis of changes in the levels of specific proteins (Fig. 7*B*) again indicated differential expression profiles between CerS6-Flag (Table 1) and CerS6^Δ338–344^-Flag-transfected cells (Table 2), with a noticeable reduction in levels of ORMDL1 in CerS6-Flag-transfected cells (Fig. 7*C*), a negative regulator of SL synthesis (23). Although changes in protein levels are to be expected after CerS6-Flag transfection, which results in changes in the SL profile (Fig. 6), changes in protein expression after transfection with CerS6^Δ338–344^-Flag are perhaps less expected because no changes were detected in the SL profile (Fig. 6). However, ORMDL1 levels were elevated in CerS6^Δ338–344^-Flag transfected cells in the proteomics analysis (Fig. 6*C*); unfortunately, validation was not possible because of the lack of antibodies able to distinguish between ORMDL isoforms. No changes were detected by qRT-PCR in the levels of ORMDL RNA for any of the isoforms, suggesting that the change in ORMDL1 levels is post-translational. Because CerS6^Δ338–344^-Flag is able to synthesize ceramide *in vitro*, but ceramide levels are unchanged in cells 48 h after over-expression, this implies that the DxRSDxE motif is nevertheless involved in the regulation of ceramide synthesis, although more precise mechanistic details remain to be determined.

**Table 1:** Top 15 hits for CerS6 over-expression obtained by analysis of Volcano plots

Gene name	Protein ID	Protein name	Ratio
CERS6	Q6ZMG9	Ceramide synthase 6	85.16
HIST1H3A	P68431	Histone H3.1	8.00
TOMM5	Q8N4H5	Mitochondrial import receptor subunit TOM5 homolog	6.77
NDUFC1	O43677	NADH dehydrogenase [ubiquinone] 1 subunit C1, mitochondrial	4.80
SSNA1	O43805	Sjogren syndrome nuclear autoantigen 1	3.54
GNG5	P63218	Guanine nucleotide-binding protein G(I)/G(S)/G(O) subunit gamma-5	3.34
NUDT16	Q96DE0	U8 snoRNA-decapping enzyme	2.96
BLOC1S1	P78537	Biogenesis of lysosome-related organelles complex 1 subunit 1	2.74
MRPL52	Q86TS9	39S ribosomal protein L52, mitochondrial	2.66
TMEM14C	Q9P0S9	Transmembrane protein 14C	2.51
SMDT1	Q9H4I9	Essential MCU regulator, mitochondrial	2.50
MAD1L1	Q9Y6D9	Mitotic spindle assembly checkpoint protein MAD1	0.38
SNUPN	O95149	Snurportin-1	0.36
MAGI1	Q96QZ7	Membrane-associated guanylate kinase, WW, and PDZ domain-containing protein 1	0.31
ORMDL1	Q9P0S3	ORM1-like protein 1	0.29

A complete list is given in Table S3. *p* < 0.05.

**Table 2:** Top 15 hits for CerS6^Δ338–344^ over-expression obtained by analysis of Volcano plots

Gene name	Protein ID	Protein name	Ratio
CERS6	Q6ZMG9	Ceramide synthase 6	62.98
TOMM5	Q8N4H5	Mitochondrial import receptor subunit TOM5 homolog	7.33
TOMM6	Q96B49	Mitochondrial import receptor subunit TOM6 homolog	5.85
NDUFC1	O43677	NADH dehydrogenase [ubiquinone] 1 subunit C1, mitochondrial	5.54
GNG5	P63218	Guanine nucleotide-binding protein G(I)/G(S)/G(O) subunit gamma-5	4.59
HIGD2A	Q9BW72	HIG1 domain family member 2A, mitochondrial	4.04
SSNA1	O43805	Sjogren syndrome nuclear autoantigen 1	3.87
COX14	Q96I36	Cytochrome c oxidase assembly protein COX14	3.50
PFDN6	O15212	Prefoldin subunit 6	3.43
NUDT16	Q96DE0	U8 snoRNA-decapping enzyme	3.13
HRNR	Q86YZ3	Hornerin	3.06
MRPL52	Q86TS9	39S ribosomal protein L52, mitochondrial	3.03
DPY30	Q9C005	Protein dpy-30 homolog	2.89
HJURP	Q8NCD3	Holliday junction recognition protein	0.24
FLG2	Q5D862	Filaggrin-2	0.23

A complete list is given in Table S3. *p* < 0.05.

## Discussion

Significant progress has been made in understanding the modes of regulation of the CerSs since their initial discovery in mammalian cells ∼20 years ago (24), largely based on bioinformatics analyses as well as site-directed mutagenesis and domain-swapping experiments (5, 10, 11, 16, 25). Various motifs and domains have been identified, with most attention paid to the Lag1p motif (13, 24, 26), likely containing the active site, and to the TLC domain in which the Lag1p motif resides. Far less attention has been paid to the C-terminus, which is likely located in the cytosol (2). The C-terminus contains a number of putative phosphorylation sites (5, 27), which can regulate CerS activity, and the C-terminus appears to be a hot-spot for splice variants (17, 18, 19). In the present study, we define a new motif at the C-terminus, found only in CerSs and not in other TLC-family members. Indeed, the presence of the DxRSDxE motif appears the most precise way of distinguishing the CerS clade from other TLC-domain containing proteins.

With the very recent availability of the alpha-fold algorithm (28) to predict 3D structures of proteins, it might have been expected that useful information about the C-terminus should now be available. However, the C-terminus of the mammalian CerS, analyzed by alpha-fold, did not yield any helpful structural information. Whether this is because of the lack of homology between the C-terminus of the different CerS, or whether this region is a genuinely unstructured region, awaits further analysis, and possibly experimental resolution of the CerS structure. Irrespective of this, no information regarding the putative role of the DxRSDxE motif can be gleaned from currently available structural data, and thus understanding the function of this motif can only be obtained by the kind of biochemical approach adopted in the present study.

Our data clearly demonstrates that deletion of the DxRSDxE motif does not affect the ability of individual CerS to synthesize ceramides. However, when the motif is deleted in two CerSs, it becomes clear that the motif is involved in regulating the activity of the other CerS, particularly CerS2, consistent with our earlier data (6). CerS2 generally has a lower activity than CerS5 or 6, as measured by analysis of kinetic parameters (5, 29), and appears more sensitive to regulation, not least *via* the DxRSDxE motif, than CerS which display higher levels of catalytic activity.

The fact that the motif is involved in the formation of CerS homo- (and hetero-) dimers is not in doubt based on immunoprecipitation studies. However, the lack of changes of ceramide and dihydroceramide levels in cultured cells upon transfection with constructs lacking the DxRSDxE motif renders it difficult to determine the precise role of this motif *in vivo*. It should be noted that this is not the first time that levels of ceramide measured in cultured cells upon deletion, or manipulation of various sequences in CerS, does not correspond to direct enzymatic assays *in vitro* (5, 11), suggesting additional and currently unknown mechanisms of CerS regulation in cells. One critical difference between the *in vitro* CerS assays and analysis of ceramide levels in cells by LC-MS/MS is that the former directly measures enzyme activity, whereas the cultured cells may be able to overcome and compensate for the presence of modified CerS sequences. Some evidence supporting this is provided by our proteomics data in as much as transfection with CerS6^Δ338–344^ did result in changes in the cellular proteome compared to both cells transfected with pcDNA or with the full length CerS6. Important among these changes was the elevation in the levels of ORMDL1, a negative regulator of SL synthesis, implying that cells sense the lack of the DxRSDxE motif and attempt to overcome the effect of this deletion.

In summary, our study has identified a novel motif which defines the CerS family and which plays a critical role in the formation of CerS dimers and thereby regulates ceramide synthesis.

## Experimental procedures

### Materials

NBD-Sphinganine and fatty acyl CoAs were from Avanti Polar Lipids. Defatted-bovine serum albumin, a protease inhibitor cocktail, anti-HA, anti-CerS2, and anti-tubulin antibodies were from Sigma-Aldrich. Protein A agarose beads and anti-CerS6 antibodies were from Santa Cruz. Horseradish peroxidase was from the Jackson Laboratory. An ECL detection system was from Cyanagen. Silica gel 60 thin layer chromatography plates were from Merck. All solvents were of analytical grade and purchased from Bio-Lab.

### Phylogenetics analysis

Sequences were chosen from the PFAM database version 33.1 (30), using the TRAM_LAG1_CLN8 (PF03798) species tree. 90 species were chosen, with a wide species diversity. All sequences from these species were taken, for a total of 766 redundant sequences. Sequences from each species were aligned separately to obtain a nonredundant set. Additional pruning was carried out upon alignment, removing sequences with large length variation (both overall and within the domain), and a set of 630 sequences was obtained. The alignments were performed with ClustalW 2.1 (31), Muscle 3.8.31 (32), and Mafft 7.480 (33) on both the full length protein sequences and on the domain sequences. Phylogenetic analysis was performed with Neighbor-Joining within ClustalW 2.1, Phylip 3.697 (34), and PhyML 3.0 (35). Additional domain assessment was performed with Prosite (https://prosite.expasy.org) (PS50922, TLC domain profile). Trees were visualized with iTOL v5 (http://itol.embl.de/) (36). The tree shown in Figure 1 is a muscle alignment with a Neighbor-Joining tree on the full length protein sequences. The CerS branch in the original tree had 244 sequences. Additional alignments of the CerS branch were performed, and the sequences with large length variations in the domain were removed, leaving 209 sequences. The Muscle alignment was used for logo visualization. Columns with less than ten sequences were removed, and logos were created with WebLogo 3 (http://weblogo.threeplusone.com) (37). Table S1 lists the sequences by clade, and in addition, indicates which sequences have the DxRSDxE motif, which were only included in the CerS alignment, and whether or not the domain was also found using ScanProsite (38).

### CerS constructs

The constructs listed in Table 3 were subcloned using restriction-free cloning (39) from CerS2 or CerS6 in a pcDNA 3.1(+) vector containing a C-terminus HA or Flag tag. The primers are given in Table 3. All sequences were confirmed before use.

**Table 3:** Primers used for subcloning

Construct	Primers	
CerS2^Δ335–380^-HA	F	GGCCCACAAGTTCATAACTGGAAAGTACCCATACGATGTTCCAGATTAC
	R	GTAATCTGGAACATCGTATGGGTACTTTCCAGTTATGAACTTGTGGGCC
CerS2^Δ338–344^-HA	F	CAAGTTCATAACTGGAAAGCTGGTAGAAGAAACAGAGAGCTCAGAGGGGGAG
	R	CTCCCCCTCTGAGCTCTCTGTTTCTTCTACCAGCTTTCCAGTTATGAACTTG
CerS2^Δ347–357^-HA	F	GAACGCAGTGACCGGGAAGAAACAGGAGGAGCAAAGAGCCGGCC
	R	GGCCGGCTCTTTGCTCCTCCTGTTTCTTCCCGGTCACTGCGTTC
CerS2^Δ358–364^-HA	F	GGGGGAGGAGGCTGCAGCTGGGCTAGCCAATGGCCACCCCATCCTCA
	R	TGAGGATGGGGTGGCCATTGGCTAGCCCAGCTGCAGCCTCCTCCCCC
CerS2^Δ365–371^-HA	F	GGGAGGAGCAAAGAGCCGGCCCCTCAATAACAACCATCGTAAGAATGAC
	R	GTCATTCTTACGATGGTTGTTATTGAGGGGCCGGCTCTTTGCTCCTCCC
CerS6^Δ335–384^-Flag	F	CTTGCAAAGCTGTTTCAAGAGGCAAGGATTACAAGGATGACGACGATAAG
	R	CTTATCGTCGTCATCCTTGTAATCCTTGCCTCTTGAAACAGCTTTGCAAG
CerS6^Δ338–344^-Flag	F	CTGTTTCAAGAGGCAAGGTGTCCAAGTCTAGCTCAGATGAGGAGGACTCAG
	R	CTGAGTCCTCCTCATCTGAGCTAGACTTGGACACCTTGCCTCTTGAAACAG
CerS6^Δ348–361^-Flag	F	CGAAGTGATATTGAGTCTAGCTCAGCGACAACCACCAATGGGACC
	R	GGTCCCATTGGTGGTTGTCGCTGAGCTAGACTCAATATCACTTCG
CerS6^Δ359–365^-Flag	F	GGACTCAGAACCTCCGGGAAAGAATAATGGGACCAGTGGTACCAACGG
	R	CCGTTGGTACCACTGGTCCCATTATTCTTTCCCGGAGGTTCTGAGTCC
CerS6^Δ366–372^-Flag	F	GAATCCCCACACTGCGACAACCACCGGGTATCTCCTGACTGGCTCCTGC
	R	GCAGGAGCCAGTCAGGAGATACCCGGTGGTTGTCGCAGTGTGGGGATTC

Abbreviations: F, forward; R, reverse.

### Cell culture and transfection

Human embryonic kidney 293T cells and CerS2 ^−/−^ Hek293T cells (11) were cultured in Dulbecco’s modified Eagle’s medium (DMEM) supplemented with 10% fetal bovine serum, 100 IU/ml penicillin, 100 μg/ml streptomycin, and 110 μg/ml sodium pyruvate. Transfections were performed with the polyethylenimine reagent (Sigma-Aldrich) using 8 μg of plasmid per 10 cm culture dish and 1.6 μg of plasmid per well in 6-well plates. The cells were removed from culture dishes after 48 h and washed twice with PBS on ice.

### Ceramide synthase assays

CerS assays were performed essentially as described (29, 40). The cells were homogenized in 20 mM Hepes–KOH, pH 7.2, 25 mM KCl, 250 mM sucrose, and 2 mM MgCl_2_ containing a protease inhibitor cocktail (Sigma-Aldrich). Protein was determined using the bicinchoninic acid assay (Cyanagen). Cell homogenates were incubated with 15 μM NBD-Sph, 20 μM defatted-bovine serum albumin, and 50 μM fatty acyl CoA in a 20 μl reaction volume at 37 °C. Reactions were terminated by the addition of chloroform/methanol (1:2, v/v) and lipids extracted (41). The lipids were dried under N_2_ and resuspended in chloroform/methanol (9:1, v/v) and applied on thin layer chromatography plates (20 × 10 cm Silica Gel plates). The thin layer chromatography plates were developed using chloroform/methanol/2 M NH_4_OH (40:10:1, v/v/v). NBD-labeled lipids were visualized using an Amersham Typhoon 5 biomolecular fluorescence imager and quantified by ImageQuantTL (GE Healthcare).

### Metabolic labeling with NBD-Sph

One milliliter of NBD-Sph (3 μM) in growth medium was added to culture dishes for 10 min (42). Cells were then placed on ice and washed twice with PBS before lipid extraction using chloroform/methanol (1:2, v/v) (41). NBD-labeled (dihydro)ceramides were separated into LC- and VLC-ceramides by TLC using chloroform/methanol/2 M NH_4_OH as the developing solvent (40:10:1, v/v/v) and quantified by ImageQuantTL (GE Healthcare). Note that this method cannot distinguish between dihydroceramides (d18:0) and ceramides (d18:1) and are therefore referred to as NBD-(dihydro)ceramides.

### Western blotting

Proteins were separated by SDS-PAGE and transferred to nitrocellulose membranes. Hemagglutinin-tagged CerS2 was immunoprecipitated using a rabbit anti-HA antibody and protein A agarose beads. CerS6 was identified using a mouse anti-CerS6 antibody (1:1000; Santa Cruz) and goat anti-mouse horseradish peroxidase (1:10,000; Jackson Laboratory) as the secondary antibody. CerS2 was detected using a rabbit anti-CerS2 antibody (1:5000; Sigma) followed by incubating with goat anti-rabbit horseradish peroxidase (1:10,000) as the secondary antibody. Equal loading was confirmed using a mouse anti-tubulin antibody (1:5000; Sigma). Detection was performed using an ECL detection system (Cyanagen).

### Coimmunoprecipitation of CerSs

Forty eight hours after transfection, the cells were washed twice with PBS and lysed in lysis buffer (150 mM NaCl, 50 mM Tris–HCl pH 7.4, 5% glycerol, 2 mM EDTA, and 1% Nonidet P-40) containing a protease inhibitor cocktail (Sigma). Cell lysates were incubated for 30 min on ice before centrifugation (12,000*g*_av_, 4 °C, 10 min). The pellet was discarded and the supernatant incubated overnight with a rabbit anti-HA antibody in a tube rotator. After incubation, 40 μl of agarose beads conjugated to protein A (Bio-Vision) were added and mixed for 2 h. Beads were collected by centrifugation and washed three times with 1 ml of lysis buffer. All procedures were performed at 4 °C. To elute the tagged proteins from the agarose beads, the beads were boiled with sample buffer (Bio-rad).

### Proteomics

Proteomics analysis was performed, as described (43). Briefly, cell pellets were lysed with 5% SDS in 50 mM Tris–HCl and were loaded onto S-Trap microcolumns (ProtiFi). The samples were eluted after digestion with trypsin (1:50, trypsin/protein, wt/wt). The samples were loaded on a split-less nanoUPLC column (Ultra Performance Liquid Chromatography, 10 kpsi nanoAcquity; Waters). The nanoUPLC was coupled online through a nanoESI (electrospray ionization) emitter (10 μm tip; New Objective) to a quadrupole orbitrap mass spectrometer (Q Exactive HFX, Thermo Scientific) using a FlexIon nanospray apparatus (Proxeon). Raw data was processed with MaxQuant v1.6.0.16. The data was analyzed with the Andromeda search engine against the SwissProt human proteome database (November 2018 version). The minimum peptide ratio was set to one, and label-free quantification was performed using unique peptides. Label-free quantification intensities were used for further calculations using Perseus v1.6.0.7. Decoy hits were filtered out, as well as proteins that were identified on the basis of modified peptides only. The label-free quantification intensities were log transformed and only proteins that had at least two valid values in at least one experimental group were used. The remaining missing values were assigned by a random low range distribution. Student’s tests were performed between the relevant groups to identify significant changes in proteins. Proteins identified with at least two peptides, with a cutoff of 1.5-fold change and *p* value 0.05 were used for further analysis. Pathway and functional analyses were performed with Metascape using the default parameters (https://metascape.org/) (44). More details can be found in Supplemental Methods.

### Lipidomics

Samples were prepared essentially as described (45). Internal standards were from Avanti Polar Lipids (d18:0/C12:0-SM, d18:0/C12:0-ceramide). Lipids were extracted and divided into two equal fractions, the single phase and organic phase (45). The pellet from the single phase was re-extracted with 1 ml methanol:chloroform (2:1 v/v). The fractions were evaporated under N_2_ and reconstituted in 150 μl of lipid mobile phase B [double-distilled water: acetonitrile: isopropanol; 1:69:30 (v/v/v)] with 1% 1 M ammonium acetate, 0.1% glacial acetic acid. The organic phase was used for analysis of ceramides and dihydroceramides. SLs were measured using a Xevo TQ-XS (Waters) mass spectrometer combined with an Acquity UPLC I class system in positive ionization mode using multiple-reaction monitoring. MS parameters were as follows: the source and desolvation temperatures were maintained at 150 °C and 400 °C, respectively. The capillary voltage was set to 2.0 kV. Nitrogen was used as the desolvation gas and cone gas at a flow rate of 800 l/h and 150 l/h, respectively. The chromatographic separation (46) was performed on an Acquity UPLC BEH C8 column (2.1 × 100 mm, internal diameter of 1.7 μm) (Waters Corp). Mobile phase A consisted of double-distilled water: acetonitrile: isopropanol; 46:38:16 (v/v/v) with 1% 1 M ammonium acetate and 0.1% glacial acetic acid. The column was maintained at 40 °C, and the flow rate of the mobile phase was 0.4 ml/min. Mobile phase A was run for 1 min and then gradually mixed with mobile phase B for an A/B ratio of 25/75 (12 min), followed by a linear gradient to 100% with mobile phase B at 16 min, and maintained till 21 min. At 21.5 min, it was restored to 100% mobile phase A and held for 3.5 min until 25 min.

### Statistics

Statistical significance was assessed using an unpaired one-tailed Student’s *t* test for CerS activity assays and for metabolic labeling with NBD-Sph. An unpaired two-tailed Student’s *t* test was used for proteomics and for ceramide measurements using mass-spectrometry. ∗*p* < 0.05; ∗∗*p* < 0.01; ∗∗∗*p* < 0.001.

## Data availability

The mass spectrometry proteomics data was deposited to the ProteomeXchange Consortium *via* the PRIDE (47) partner repository with the dataset identifier PXD029496. The data that support the findings of this study are available from the corresponding author upon reasonable request.

## Supporting information

This article contains supporting information

## Conflict of interest

The authors declare that they have no conflicts of interest with the contents of this article.

## Supporting information

Supplemental Table S1

Supplemental Table S2 Complete list of identified proteins by proteomics analysis.

Supplemental Table S3

Supplemental Methods Additional methods regarding proteomics.
