# Supplementary material for: A novel C-terminal DxRSDxE motif in ceramide synthases involved in dimer formation
Source: J Biol Chem. 2021 Dec 20;298(2):101517. doi: 10.1016/j.jbc.2021.101517 (PMC8789539; doi:10.1016/j.jbc.2021.101517)
Supplement: Supplemental Table S3 [file mmc3.docx]

**Supporting Information**

**Supplementary Table 3. Differentially-expressed proteins in Hek293T cells transfected with Flag-tagged CerS6 or CerS6 ^Δ338-344^.**

|  |  |  | **CerS6:pcDNA** | | | **CerS6 ^Δ338-344^**  **:pcDNA** | | | **CerS6**  **:CerS6 ^Δ338-344^** | |
| --- | --- | --- | --- | --- | --- | --- | --- | --- | --- | --- |
| Protein ID | Protein name | Gene name | | *p-value* | Ratio | | *p-value* | Ratio | *p-value* | Ratio |
| Q6ZMG9 | Ceramide synthase 6 | CERS6 | | <0.01 | 85.2 | | <0.01 | 63.0 | 0.094 | 1.35 |
| P68431 | Histone H3.1 | HIST1H3A | | 0.028 | 8.00 | | 0.28 | 5.07 | 0.771 | 1.58 |
| Q8N4H5 | Mitochondrial import receptor subunit TOM5 homolog | TOMM5 | | 0.013 | 6.77 | | 0.018 | 7.33 | 0.822 | 0.92 |
| O43677 | NADH dehydrogenase [ubiquinone] 1 subunit C1, mitochondrial | NDUFC1 | | 0.019 | 4.80 | | 0.016 | 5.54 | 0.504 | 0.87 |
| O43805 | Sjoegren syndrome nuclear autoantigen 1 | SSNA1 | | 0.015 | 3.54 | | 0.013 | 3.87 | 0.695 | 0.91 |
| P63218 | Guanine nucleotide-binding protein G(I)/G(S)/G(O) subunit gamma-5 | GNG5 | | 0.038 | 3.34 | | 0.025 | 4.59 | 0.322 | 0.73 |
| Q96DE0 | U8 snoRNA-decapping enzyme | NUDT16 | | <0.01 | 2.96 | | <0.01 | 3.13 | 0.658 | 0.94 |
| P78537 | Biogenesis of lysosome-related organelles complex 1 subunit 1 | BLOC1S1 | | 0.044 | 2.74 | | 0.052 | 2.94 | 0.761 | 0.93 |
| Q86TS9 | 39S ribosomal protein L52, mitochondrial | MRPL52 | | 0.002 | 2.66 | | 0.01 | 3.03 | 0.623 | 0.88 |
| Q9P0S9 | Transmembrane protein 14C | TMEM14C | | 0.030 | 2.51 | | 0.042 | 2.83 | 0.772 | 0.89 |
| Q9H4I9 | Essential MCU regulator, mitochondrial | SMDT1 | | 0.040 | 2.50 | | 0.100 | 2.84 | 0.790 | 0.88 |
| P18859 | ATP synthase-coupling factor 6, mitochondrial | ATP5J | | 0.011 | 2.30 | | 0.018 | 2.40 | 0.798 | 0.96 |
| O15212 | Prefoldin subunit 6 | PFDN6 | | 0.009 | 2.30 | | 0.003 | 3.43 | 0.152 | 0.67 |
| Q9Y2R0 | Cytochrome c oxidase assembly factor 3 homolog, mitochondrial | COA3 | | 0.005 | 2.28 | | 0.03 | 2.55 | 0.720 | 0.90 |
| Q9NU23 | LYR motif-containing protein 2 | LYRM2 | | 0.019 | 2.27 | | 0.025 | 2.26 | 0.962 | 1.01 |
| O75954 | Tetraspanin-9 | TSPAN9 | | 0.026 | 2.25 | | 0.125 | 1.66 | 0.363 | 1.36 |
| Q70HW3 | S-adenosylmethionine mitochondrial carrier protein | SLC25A26 | | 0.015 | 2.23 | | 0.499 | 1.29 | 0.115 | 1.73 |
| Q9UBI6 | Guanine nucleotide-binding protein G(I)/G(S)/G(O) subunit gamma-12 | GNG12 | | 0.026 | 2.15 | | 0.015 | 2.55 | 0.181 | 0.85 |
| O75438 | NADH dehydrogenase [ubiquinone] 1 beta subcomplex subunit 1 | NDUFB1 | | 0.001 | 2.00 | | 0.023 | 2.16 | 0.765 | 0.93 |
| P14406 | Cytochrome c oxidase subunit 7A2, mitochondrial | COX7A2 | | 0.004 | 1.98 | | 0.005 | 2.42 | 0.292 | 0.82 |
| Q9UDW1 | Cytochrome b-c1 complex subunit 9 | UQCR10 | | 0.004 | 1.98 | | 0.028 | 1.87 | 0.773 | 1.06 |
| Q9Y5J6 | Mitochondrial import inner membrane translocase subunit Tim10 B | TIMM10B | | 0.005 | 1.97 | | 0.006 | 2.51 | 0.299 | 0.78 |
| Q9Y3E0 | Vesicle transport protein GOT1B | GOLT1B | | 0.004 | 1.95 | | 0.044 | 1.41 | 0.044 | 1.39 |
| P56385 | ATP synthase subunit e, mitochondrial | ATP5I | | 0.010 | 1.92 | | 0.006 | 2.11 | 0.325 | 0.91 |
| P00156 | Cytochrome b | MT-CYB | | 0.002 | 1.92 | | 0.328 | 1.51 | 0.577 | 1.27 |
| Q14410 | Glycerol kinase 2 | GK2 | | 0.036 | 1.91 | | 0.065 | 2.07 | 0.748 | 0.92 |
| O60262 | Guanine nucleotide-binding protein G(I)/G(S)/G(O) subunit gamma-7 | GNG7 | | 0.026 | 1.88 | | 0.152 | 1.96 | 0.909 | 0.96 |
| Q9NR77 | Peroxisomal membrane protein 2 | PXMP2 | | <0.01 | 1.82 | | 0.095 | 1.55 | 0.484 | 1.18 |
| O95295 | SNARE-associated protein Snapin | SNAPIN | | 0.032 | 1.78 | | <0.01 | 2.27 | 0.060 | 0.79 |
| O60925 | Prefoldin subunit 1 | PFDN1 | | 0.020 | 1.77 | | 0.033 | 1.82 | 0.853 | 0.97 |
| Q9Y5L4 | Mitochondrial import inner membrane translocase subunit Tim13 | TIMM13 | | 0.020 | 1.75 | | 0.010 | 1.90 | 0.473 | 0.92 |
| P23025 | DNA repair protein complementing XP-A cells | XPA | | <0.01 | 1.73 | | 0.064 | 1.50 | 0.434 | 1.15 |
| Q9P0U1 | Mitochondrial import receptor subunit TOM7 homolog | TOMM7 | | 0.014 | 1.73 | | <0.01 | 2.16 | <0.01 | 0.80 |
| Q8WUW1 | Protein BRICK1 | BRK1 | | 0.035 | 1.72 | | 0.006 | 2.78 | 0.134 | 0.62 |
| Q15629 | Translocating chain-associated membrane protein 1 | TRAM1 | | <0.01 | 1.72 | | 0.039 | 1.65 | 0.840 | 1.04 |
| Q96IX5 | Up-regulated during skeletal muscle growth protein 5 | USMG5 | | <0.01 | 1.72 | | 0.028 | 1.65 | 0.745 | 1.04 |
| Q6N075 | Molybdate-anion transporter | MFSD5 | | <0.01 | 1.71 | | 0.177 | 1.28 | 0.114 | 1.34 |
| Q8N5G0 | Small integral membrane protein 20 | SMIM20 | | <0.01 | 1.70 | | 0.015 | 1.71 | 0.95 | 0.99 |
| Q9NR28 | Diablo homolog, mitochondrial | DIABLO | | <0.01 | 1.70 | | 0.028 | 1.59 | 0.669 | 1.07 |
| O95476 | CTD nuclear envelope phosphatase 1 | CTDNEP1 | | 0.015 | 1.69 | | 0.785 | 0.93 | 0.040 | 1.83 |
| Q9H4B0 | Probable tRNA N6-adenosine threonylcarbamoyltransferase, mitochondrial | OSGEPL1 | | 0.017 | 1.69 | | 0.116 | 1.63 | 0.912 | 1.03 |
| P84157 | Matrix-remodeling-associated protein 7 | MXRA7 | | <0.01 | 1.67 | | <0.01 | 2.17 | 0.227 | 0.77 |
| Q9NYJ1 | Cytochrome c oxidase assembly factor 4 homolog, mitochondrial | COA4 | | 0.012 | 1.62 | | <0.01 | 1.93 | 0.114 | 0.84 |
| O43826 | Glucose-6-phosphate translocase | SLC37A4 | | <0.01 | 1.61 | | 0.011 | 1.52 | 0.596 | 1.06 |
| Q99595 | Mitochondrial import inner membrane translocase subunit Tim17-A | TIMM17A | | <0.01 | 1.61 | | 0.015 | 1.55 | 0.641 | 1.04 |
| Q96CQ1 | Solute carrier family 25 member 36 | SLC25A36 | | 0.018 | 1.60 | | 0.035 | 1.49 | 0.387 | 1.07 |
| Q92674 | Centromere protein I | CENPI | | 0.025 | 1.60 | | 0.75 | 0.92 | 0.036 | 1.74 |
| Q9Y5J9 | Mitochondrial import inner membrane translocase subunit Tim8 B | TIMM8B | | 0.042 | 1.59 | | 0.02 | 1.82 | 0.252 | 0.87 |
| P10620 | Microsomal glutathione S-transferase 1 | MGST1 | | <0.01 | 1.59 | | 0.103 | 1.48 | 0.714 | 1.08 |
| P09669 | Cytochrome c oxidase subunit 6C | COX6C | | 0.013 | 1.59 | | <0.01 | 1.86 | 0.316 | 0.85 |
| O95168 | NADH dehydrogenase [ubiquinone] 1 beta subcomplex subunit 4 | NDUFB4 | | <0.01 | 1.59 | | 0.014 | 1.65 | 0.702 | 0.96 |
| Q9H4I3 | TraB domain-containing protein | TRABD | | 0.035 | 1.58 | | 0.087 | 1.57 | 0.945 | 1.01 |
| Q7KZN9 | Cytochrome c oxidase assembly protein COX15 homolog | COX15 | | <0.01 | 1.57 | | 0.078 | 1.47 | 0.722 | 1.07 |
| Q86WP2 | Vasculin | GPBP1 | | 0.036 | 1.56 | | 0.07 | 1.52 | 0.877 | 1.02 |
| P00846 | ATP synthase subunit a | MT-ATP6 | | <0.01 | 1.56 | | 0.077 | 1.53 | 0.939 | 1.02 |
| Q15800 | Methylsterol monooxygenase 1 | MSMO1 | | <0.01 | 1.55 | | 0.124 | 1.29 | 0.113 | 1.21 |
| O15514 | DNA-directed RNA polymerase II subunit RPB4 | POLR2D | | 0.014 | 1.55 | | 0.022 | 1.83 | 0.438 | 0.85 |
| Q9NS69 | Mitochondrial import receptor subunit TOM22 homolog | TOMM22 | | 0.041 | 1.55 | | 0.03 | 1.74 | 0.422 | 0.89 |
| Q99471 | Prefoldin subunit 5 | PFDN5 | | 0.017 | 1.54 | | 0.008 | 1.69 | 0.222 | 0.92 |
| L0R6Q1 |  | SLC35A4 | | 0.030 | 1.54 | | 0.030 | 1.60 | 0.750 | 0.96 |
| P15954 | Cytochrome c oxidase subunit 7C, mitochondrial | COX7C | | 0.011 | 1.54 | | <0.01 | 1.75 | 0.187 | 0.88 |
| Q14442 | Phosphatidylinositol N-acetylglucosaminyltransferase subunit H | PIGH | | 0.040 | 1.53 | | 0.497 | 1.19 | 0.294 | 1.29 |
| O95807 | Transmembrane protein 50A | TMEM50A | | 0.049 | 1.53 | | 0.036 | 1.53 | 0.997 | 1.00 |
| O14880 | Microsomal glutathione S-transferase 3 | MGST3 | | 0.037 | 1.53 | | 0.302 | 1.57 | 0.949 | 0.98 |
| O14521 | Succinate dehydrogenase [ubiquinone] cytochrome b small subunit, mitochondrial | SDHD | | 0.035 | 1.52 | | 0.649 | 1.20 | 0.522 | 1.27 |
| Q9Y5J7 | Mitochondrial import inner membrane translocase subunit Tim9 | TIMM9 | | 0.016 | 1.51 | | 0.031 | 1.61 | 0.665 | 0.94 |
| Q96BP2 | Coiled-coil-helix-coiled-coil-helix domain-containing protein 1 | CHCHD1 | | 0.028 | 1.51 | | 0.018 | 1.68 | 0.294 | 0.90 |
| Q16718 | NADH dehydrogenase [ubiquinone] 1 alpha subcomplex subunit 5 | NDUFA5 | | 0.015 | 1.51 | | 0.026 | 1.46 | 0.738 | 1.03 |
| Q99643 | Succinate dehydrogenase cytochrome b560 subunit, mitochondrial | SDHC | | <0.01 | 1.50 | | 0.071 | 1.38 | 0.551 | 1.09 |
| Q9BWJ5 | Splicing factor 3B subunit 5 | SF3B5 | | 0.034 | 1.50 | | 0.096 | 1.78 | 0.555 | 0.84 |
| Q9NVV5 | Androgen-induced gene 1 protein | AIG1 | | 0.025 | 1.50 | | <0.01 | 1.70 | 0.398 | 0.88 |
| P61619 | Protein transport protein Sec61 subunit alpha isoform 1 | SEC61A1 | | 0.034 | 1.50 | | 0.017 | 1.66 | 0.613 | 0.90 |
| P13498 | Cytochrome b-245 light chain | CYBA | | 0.025 | 1.50 | | 0.615 | 1.11 | 0.096 | 1.34 |
| O95671 | N-acetylserotonin O-methyltransferase-like protein | ASMTL | | 0.023 | 0.66 | | 0.393 | 1.20 | 0.022 | 0.55 |
| Q5VYS8 | Terminal uridylyltransferase 7 | ZCCHC6 | | 0.039 | 0.66 | | 0.491 | 0.89 | 0.032 | 0.74 |
| P42166 | Lamina-associated polypeptide 2, isoform alpha;Thymopoietin;Thymopentin | TMPO | | <0.01 | 0.66 | | 0.012 | 0.71 | 0.426 | 0.93 |
| Q99666 | RANBP2-like and GRIP domain-containing protein 5/6 | RGPD5 | | 0.011 | 0.66 | | 0.393 | 0.87 | 0.027 | 0.75 |
| O94806 | Serine/threonine-protein kinase D3 | PRKD3 | | 0.015 | 0.65 | | 0.634 | 1.08 | 0.029 | 0.60 |
| P37275 | Zinc finger E-box-binding homeobox 1 | ZEB1 | | <0.01 | 0.64 | | 0.096 | 0.73 | 0.482 | 0.89 |
| Q6VMQ6 | Activating transcription factor 7-interacting protein 1 | ATF7IP | | <0.01 | 0.64 | | 0.060 | 0.72 | 0.399 | 0.89 |
| Q9BQS8 | FYVE and coiled-coil domain-containing protein 1 | FYCO1 | | <0.01 | 0.64 | | 0.011 | 0.64 | 0.928 | 0.99 |
| P12270 | Nucleoprotein TPR | TPR | | <0.01 | 0.63 | | 0.014 | 0.63 | 0.918 | 1.01 |
| Q8IWJ2 | GRIP and coiled-coil domain-containing protein 2 | GCC2 | | <0.01 | 0.63 | | 0.012 | 0.74 | 0.149 | 0.85 |
| Q15013 | MAD2L1-binding protein | MAD2L1BP | | 0.0238 | 0.63 | | 0.741 | 0.91 | 0.172 | 0.69 |
| Q14149 | MORC family CW-type zinc finger protein 3 | MORC3 | | 0.0263 | 0.63 | | 0.480 | 0.84 | 0.215 | 0.75 |
| Q9Y2V7 | Conserved oligomeric Golgi complex subunit 6 | COG6 | | <0.01 | 0.62 | | <0.01 | 0.59 | 0.579 | 1.05 |
| O94913 | Pre-mRNA cleavage complex 2 protein Pcf11 | PCF11 | | <0.01 | 0.62 | | 0.049 | 0.72 | 0.312 | 0.87 |
| Q9P2D3 | HEAT repeat-containing protein 5B | HEATR5B | | 0.012 | 0.62 | | 0.307 | 0.82 | 0.091 | 0.76 |
| E9PRG8 | Uncharacterized protein C11orf98 | C11orf98 | | 0.010 | 0.62 | | 0.055 | 0.79 | 0.113 | 0.79 |
| P61956 | Small ubiquitin-related modifier 2 | SUMO2 | | 0.030 | 0.62 | | 0.188 | 0.74 | 0.207 | 0.84 |
| Q9UBW7 | Zinc finger MYM-type protein 2 | ZMYM2 | | <0.01 | 0.62 | | 0.018 | 0.66 | 0.647 | 0.94 |
| Q86WB0 | Nuclear-interacting partner of ALK | ZC3HC1 | | <0.01 | 0.61 | | 0.025 | 0.66 | 0.478 | 0.91 |
| Q9NQ75 | Cas scaffolding protein family member 4 | CASS4 | | 0.048 | 0.60 | | 0.664 | 0.79 | 0.605 | 0.77 |
| P83436 | Conserved oligomeric Golgi complex subunit 7 | COG7 | | 0.010 | 0.60 | | 0.009 | 0.63 | 0.818 | 0.96 |
| Q16656 | Nuclear respiratory factor 1 | NRF1 | | 0.005 | 0.60 | | 0.030 | 0.79 | 0.088 | 0.76 |
| Q13439 | Golgin subfamily A member 4 | GOLGA4 | | 0.012 | 0.60 | | 0.036 | 0.67 | 0.561 | 0.89 |
| Q9H9E3 | Conserved oligomeric Golgi complex subunit 4 | COG4 | | 0.016 | 0.58 | | 0.162 | 0.73 | 0.304 | 0.80 |
| Q8N0T1 | Uncharacterized protein C8orf59 | C8orf59 | | <0.01 | 0.58 | | 0.039 | 0.77 | 0.042 | 0.75 |
| Q9UKY1 | Zinc fingers and homeoboxes protein 1 | ZHX1 | | <0.01 | 0.58 | | 0.013 | 0.53 | 0.690 | 1.08 |
| Q9Y2G3 | Probable phospholipid-transporting ATPase IF | ATP11B | | <0.01 | 0.57 | | 0.650 | 1.19 | 0.086 | 0.48 |
| Q96MW5 | Conserved oligomeric Golgi complex subunit 8 | COG8 | | <0.01 | 0.56 | | 0.089 | 0.71 | 0.188 | 0.80 |
| Q9H019 | Mitochondrial fission regulator 1-like | MTFR1L | | 0.0105 | 0.56 | | 0.618 | 1.09 | <0.01 | 0.51 |
| Q15047 | Histone-lysine N-methyltransferase SETDB1 | SETDB1 | | <0.01 | 0.54 | | 0.016 | 0.67 | 0.120 | 0.81 |
| Q99856 | AT-rich interactive domain-containing protein 3A | ARID3A | | <0.01 | 0.53 | | 0.105 | 0.63 | 0.527 | 0.85 |
| Q8WXW3 | Progesterone-induced-blocking factor 1 | PIBF1 | | 0.017 | 0.52 | | 0.194 | 0.57 | 0.846 | 0.92 |
| O15530 | 3-phosphoinositide-dependent protein kinase 1 | PDPK1 | | 0.035 | 0.51 | | 0.802 | 0.94 | 0.113 | 0.54 |
| Q9Y6X8 | Zinc fingers and homeoboxes protein 2 | ZHX2 | | <0.01 | 0.50 | | 0.042 | 0.64 | 0.120 | 0.79 |
| Q6ZMI0 | Protein phosphatase 1 regulatory subunit 21 | PPP1R21 | | <0.01 | 0.50 | | 0.171 | 0.67 | 0.374 | 0.74 |
| Q9Y6X4 | Soluble lamin-associated protein of 75 kDa | FAM169A | | <0.01 | 0.50 | | 0.033 | 0.54 | 0.706 | 0.93 |
| Q9H4I2 | Zinc fingers and homeoboxes protein 3 | ZHX3 | | <0.01 | 0.47 | | 0.019 | 0.70 | 0.071 | 0.67 |
| Q9UP83 | Conserved oligomeric Golgi complex subunit 5 | COG5 | | 0.016 | 0.47 | | 0.157 | 0.69 | 0.216 | 0.68 |
| Q9H9L4 | KAT8 regulatory NSL complex subunit 2 | KANSL2 | | 0.032 | 0.46 | | 0.842 | 1.10 | 0.087 | 0.42 |
| Q9Y6D9 | Mitotic spindle assembly checkpoint protein MAD1 | MAD1L1 | | <0.01 | 0.38 | | <0.01 | 0.46 | 0.187 | 0.82 |
| O95149 | Snurportin-1 | SNUPN | | 0.045 | 0.36 | | 0.372 | 0.55 | 0.567 | 0.64 |
| Q96QZ7 | Membrane-associated guanylate kinase, WW and PDZ domain-containing protein 1 | MAGI1 | | 0.040 | 0.31 | | <0.01 | 0.76 | 0.093 | 0.41 |
| Q9P0S3 | ORM1-like protein 1 | ORMDL1 | | 0.019 | 0.29 | | 0.105 | 2.20 | <0.01 | 0.13 |
| Q96B49 | Mitochondrial import receptor subunit TOM6 homolog | TOMM6 | | 0.476 | 1.82 | | 0.022 | 5.85 | 0.141 | 0.31 |
| Q9BW72 | HIG1 domain family member 2A, mitochondrial | HIGD2A | | 0.120 | 1.44 | | 0.001 | 4.04 | 0.003 | 0.36 |
| Q96I36 | Cytochrome c oxidase assembly protein COX14 | COX14 | | 0.164 | 2.33 | | 0.011 | 3.50 | 0.417 | 0.67 |
| Q86YZ3 | Hornerin | HRNR | | 0.492 | 1.30 | | 0.024 | 3.06 | 0.110 | 0.43 |
| Q9C005 | Protein dpy-30 homolog | DPY30 | | 0.057 | 1.62 | | 0.018 | 2.89 | 0.107 | 0.56 |
| P35527 | Keratin, type I cytoskeletal 9 | KRT9 | | 0.570 | 1.22 | | 0.011 | 2.85 | 0.069 | 0.43 |
| Q9BXH1 | Bcl-2-binding component 3 | BBC3 | | 0.796 | 1.14 | | 0.033 | 2.82 | 0.056 | 0.41 |
| Q15121 | Astrocytic phosphoprotein PEA-15 | PEA15 | | 0.547 | 1.07 | | 0.002 | 2.69 | <0.01 | 0.40 |
| P04264 | Keratin, type II cytoskeletal 1 | KRT1 | | 0.536 | 1.43 | | 0.004 | 2.66 | 0.319 | 0.54 |
| Q5BKU9 | Oxidoreductase-like domain-containing protein 1 | OXLD1 | | 0.516 | 0.61 | | 0.032 | 2.47 | 0.085 | 0.25 |
| Q8NI22 | Multiple coagulation factor deficiency protein 2 | MCFD2 | | 0.190 | 1.28 | | 0.042 | 2.46 | 0.081 | 0.52 |
| Q9Y605 | MORF4 family-associated protein 1 | MRFAP1 | | 0.142 | 1.39 | | 0.023 | 2.39 | 0.056 | 0.58 |
| O43708 | Maleylacetoacetate isomerase | GSTZ1 | | 0.190 | 1.36 | | 0.015 | 2.37 | 0.055 | 0.57 |
| L0R8F8 |  | SMCR7L | | 0.126 | 1.63 | | 0.037 | 2.24 | 0.141 | 0.73 |
| Q9BQ87 | F-box-like/WD repeat-containing protein TBL1Y | TBL1Y | | 0.341 | 1.32 | | 0.014 | 2.23 | 0.104 | 0.59 |
| Q15528 | Mediator of RNA polymerase II transcription subunit 22 | MED22 | | 0.079 | 1.60 | | 0.021 | 2.10 | 0.079 | 0.76 |
| Q9UFG5 | UPF0449 protein C19orf25 | C19orf25 | | 0.100 | 1.48 | | 0.038 | 2.07 | 0.190 | 0.72 |
| Q9Y241 | HIG1 domain family member 1A, mitochondrial | HIGD1A | | 0.310 | 1.20 | | 0.025 | 2.04 | 0.070 | 0.59 |
| Q9UL18 | Protein argonaute-1 | AGO1 | | 0.524 | 0.87 | | 0.047 | 2.02 | <0.01 | 0.43 |
| P13647 | Keratin, type II cytoskeletal 5 | KRT5 | | 0.724 | 1.27 | | 0.039 | 2.01 | 0.490 | 0.63 |
| Q8N300 | Coiled-coil domain-containing protein 23 | CCDC23 | | 0.011 | 1.48 | | 0.012 | 1.97 | 0.134 | 0.75 |
| P60763 | Ras-related C3 botulinum toxin substrate 3 | RAC3 | | 0.982 | 1.00 | | <0.01 | 1.95 | 0.013 | 0.52 |
| O60739 | Eukaryotic translation initiation factor 1b | EIF1B | | 0.566 | 0.81 | | <0.01 | 1.92 | 0.047 | 0.42 |
| Q9GZU7 | Carboxy-terminal domain RNA polymerase II polypeptide A small phosphatase 1 | CTDSP1 | | 0.616 | 1.11 | | 0.025 | 1.90 | 0.097 | 0.59 |
| O60220 | Mitochondrial import inner membrane translocase subunit Tim8 A | TIMM8A | | 0.028 | 1.49 | | 0.022 | 1.88 | 0.214 | 0.79 |
| Q86Y79 | Probable peptidyl-tRNA hydrolase | PTRH1 | | 0.216 | 1.19 | | <0.01 | 1.88 | <0.01 | 0.63 |
| Q9Y3C7 | Mediator of RNA polymerase II transcription subunit 31 | MED31 | | 0.068 | 1.54 | | 0.015 | 1.85 | 0.068 | 0.83 |
| Q9NX70 | Mediator of RNA polymerase II transcription subunit 29 | MED29 | | 0.182 | 1.32 | | 0.019 | 1.85 | 0.062 | 0.71 |
| O43715 | TP53-regulated inhibitor of apoptosis 1 | TRIAP1 | | 0.065 | 1.51 | | 0.030 | 1.85 | 0.337 | 0.82 |
| Q9NWU2 | Glucose-induced degradation protein 8 homolog | GID8 | | 0.030 | 1.36 | | <0.01 | 1.82 | 0.021 | 0.74 |
| P62306 | Small nuclear ribonucleoprotein F | SNRPF | | 0.042 | 1.41 | | 0.016 | 1.80 | 0.169 | 0.78 |
| Q9UI95 | Mitotic spindle assembly checkpoint protein MAD2B | MAD2L2 | | 0.065 | 1.22 | | 0.015 | 1.80 | 0.073 | 0.68 |
| P60059 | Protein transport protein Sec61 subunit gamma | SEC61G | | 0.024 | 1.30 | | <0.01 | 1.79 | 0.017 | 0.73 |
| Q8N5M4 | Tetratricopeptide repeat protein 9C | TTC9C | | 0.947 | 1.01 | | <0.01 | 1.76 | 0.025 | 0.57 |
| P62304 | Small nuclear ribonucleoprotein E | SNRPE | | 0.067 | 1.21 | | <0.01 | 1.74 | 0.029 | 0.70 |
| Q9P086 | Mediator of RNA polymerase II transcription subunit 11 | MED11 | | 0.090 | 1.40 | | 0.037 | 1.73 | 0.139 | 0.80 |
| P63313 | Thymosin beta-10 | TMSB10 | | 0.187 | 1.29 | | 0.049 | 1.71 | 0.153 | 0.75 |
| O14681 | Etoposide-induced protein 2.4 homolog | EI24 | | 0.036 | 1.41 | | 0.036 | 1.66 | 0.432 | 0.85 |
| Q9HD34 | LYR motif-containing protein 4 | LYRM4 | | 0.030 | 1.42 | | 0.010 | 1.61 | 0.140 | 0.88 |
| Q15526 | Surfeit locus protein 1 | SURF1 | | 0.040 | 1.39 | | 0.038 | 1.59 | 0.430 | 0.87 |
| P31949 | Protein S100-A11;Protein S100-A11, N-terminally processed | S100A11 | | 0.189 | 1.20 | | 0.025 | 1.58 | 0.141 | 0.76 |
| O95229 | ZW10 interactor | ZWINT | | 0.325 | 1.16 | | 0.013 | 1.58 | 0.022 | 0.73 |
| Q4G0I0 | Protein CCSMST1 | CCSMST1 | | 0.406 | 1.18 | | 0.039 | 1.58 | 0.101 | 0.75 |
| P48200 | Iron-responsive element-binding protein 2 | IREB2 | | 0.581 | 0.94 | | 0.025 | 1.57 | 0.006 | 0.60 |
| P62072 | Mitochondrial import inner membrane translocase subunit Tim10 | TIMM10 | | 0.017 | 1.40 | | 0.011 | 1.55 | 0.204 | 0.90 |
| Q9H2D1 | Mitochondrial folate transporter/carrier | SLC25A32 | | 0.000 | 1.39 | | 0.009 | 1.54 | 0.394 | 0.91 |
| P05386 | 60S acidic ribosomal protein P1 | RPLP1 | | 0.876 | 1.03 | | 0.039 | 1.52 | 0.032 | 0.68 |
| P07108 | Acyl-CoA-binding protein | DBI | | 0.133 | 1.25 | | 0.017 | 1.52 | <0.01 | 0.82 |
| O14561 | Acyl carrier protein, mitochondrial | NDUFAB1 | | 0.024 | 1.34 | | 0.027 | 1.51 | 0.365 | 0.88 |
| Q96GE9 | Transmembrane protein 261 | TMEM261 | | 0.208 | 1.16 | | 0.029 | 1.51 | 0.060 | 0.77 |
| P60903 | Protein S100-A10 | S100A10 | | 0.642 | 0.94 | | 0.036 | 1.50 | 0.010 | 0.63 |
| O43678 | NADH dehydrogenase [ubiquinone] 1 alpha subcomplex subunit 2 | NDUFA2 | | 0.035 | 1.27 | | <0.01 | 1.50 | 0.004 | 0.85 |
| P41567 | Eukaryotic translation initiation factor 1 | EIF1 | | 0.077 | 1.30 | | 0.012 | 1.50 | 0.228 | 0.87 |
| Q8WTV0 | Scavenger receptor class B member 1 | SCARB1 | | 0.018 | 1.20 | | 0.017 | 1.50 | 0.097 | 0.80 |
| O95167 | NADH dehydrogenase [ubiquinone] 1 alpha subcomplex subunit 3 | NDUFA3 | | 0.041 | 1.29 | | 0.011 | 1.50 | 0.063 | 0.86 |
| Q3ZAQ7 | Vacuolar ATPase assembly integral membrane protein VMA21 | VMA21 | | 0.010 | 1.36 | | 0.013 | 1.50 | 0.384 | 0.91 |
| Q9BWH6 | RNA polymerase II-associated protein 1 | RPAP1 | | 0.074 | 0.69 | | 0.031 | 0.66 | 0.819 | 1.05 |
| O75449 | Katanin p60 ATPase-containing subunit A1 | KATNA1 | | 0.232 | 0.82 | | 0.025 | 0.66 | 0.259 | 1.24 |
| Q04446 | 1,4-alpha-glucan-branching enzyme | GBE1 | | 0.124 | 0.81 | | 0.014 | 0.65 | 0.003 | 1.24 |
| Q86XZ4 | Spermatogenesis-associated serine-rich protein 2 | SPATS2 | | 0.097 | 0.76 | | 0.049 | 0.65 | 0.400 | 1.17 |
| Q8IUI8 | Cytokine receptor-like factor 3 | CRLF3 | | 0.138 | 0.76 | | 0.032 | 0.65 | 0.036 | 1.18 |
| Q9NSC2 | Sal-like protein 1 | SALL1 | | 0.286 | 0.82 | | 0.034 | 0.64 | 0.015 | 1.28 |
| Q9HCE5 | N6-adenosine-methyltransferase subunit METTL14 | METTL14 | | 0.284 | 0.85 | | 0.030 | 0.63 | 0.122 | 1.36 |
| Q8TDY2 | RB1-inducible coiled-coil protein 1 | RB1CC1 | | 0.145 | 0.69 | | 0.028 | 0.62 | 0.594 | 1.11 |
| Q9Y2S6 | Translation machinery-associated protein 7 | TMA7 | | <0.01 | 0.70 | | 0.026 | 0.60 | 0.369 | 1.17 |
| P42684 | Abelson tyrosine-protein kinase 2 | ABL2 | | <0.01 | 0.68 | | <0.01 | 0.59 | 0.191 | 1.16 |
| Q9NPA5 | Zinc finger protein 64 homolog, isoforms 1 and 2 | ZFP64 | | 0.057 | 0.48 | | <0.01 | 0.57 | 0.584 | 0.85 |
| Q9BZG8 | Diphthamide biosynthesis protein 1 | DPH1 | | 0.141 | 0.71 | | 0.017 | 0.57 | 0.224 | 1.25 |
| Q8NCD3 | Holliday junction recognition protein | HJURP | | 0.071 | 0.52 | | <0.01 | 0.24 | 0.064 | 2.18 |
| Q5D862 | Filaggrin-2 | FLG2 | | 0.888 | 0.87 | | 0.029 | 0.23 | 0.214 | 3.73 |
