# Supplementary material for: A novel C-terminal DxRSDxE motif in ceramide synthases involved in dimer formation
Source: J Biol Chem. 2021 Dec 20;298(2):101517. doi: 10.1016/j.jbc.2021.101517 (PMC8789539; doi:10.1016/j.jbc.2021.101517)
Supplement: Supplemental Methods — Additional methods regarding proteomics. [file mmc4.docx]

**Supplemental Methods**

**Sample preparation**

Cell pellets of 4 biological replicates were lysed with 5% SDS in 50 mM Tris-HCl. Lysates were incubated at 96°C for 5 min, followed by six cycles of 30 sec of sonication (Bioruptor Pico, Diagenode, USA). Protein concentration was measured using the BCA assay (Thermo Scientific, USA) and a total of 20 μg protein was reduced with 5 mM dithiothreitol and alkylated with 10 mM iodoacetamide in the dark. Each sample was loaded onto S-Trap microcolumns (Protifi, USA) according to the manufacturer’s instructions. In brief, after loading, samples were washed with 90:10% methanol/50 mM ammonium bicarbonate. Samples were then digested with trypsin (1:50 trypsin/protein) for 1.5 h at 47°C. The digested peptides were eluted using 50 mM ammonium bicarbonate; trypsin was added to this fraction and incubated overnight at 37°C. Two more elutions were made using 0.2% formic acid and 0.2% formic acid in 50% acetonitrile. The three elutions were pooled together and vacuum-centrifuged to dry. Samples were kept at −80°C until analysis.

**Liquid chromatography**

ULC/MS grade solvents were used for all chromatographic steps. Each sample was loaded using split-less nano-Ultra Performance Liquid Chromatography (10 kpsi nanoAcquity; Waters, Milford, MA, USA). The mobile phase was: A) H2O + 0.1% formic acid and B) acetonitrile + 0.1% formic acid. Desalting of the samples was performed online using a reversed-phase Symmetry C18 trapping column (180 µm internal diameter, 20 mm length, 5 µm particle size; Waters). Peptides were separated using a T3 HSS nano-column (75 µm internal diameter, 250 mm length, 1.8 µm particle size; Waters) at 0.35 µL/min. Peptides were eluted from the column into the mass spectrometer using the following gradient: 4% to 27%B in 155 min, 27% to 90%B in 5 min, maintained at 90%B for 5 min and then back to initial conditions.

**Mass Spectrometry**

The nanoUPLC was coupled online through a nanoESI emitter (10 μm tip; New Objective; Woburn, MA, USA) to a quadrupole orbitrap mass spectrometer (Q Exactive HFX, Thermo Scientific) using a FlexIon nanospray apparatus (Proxeon). Data was acquired in data dependent acquisition (DDA) mode, using a Top10 method. MS1 resolution was set to 120,000 (at 200 m/z), mass range of 375-1650 m/z, AGC of 3e6 and maximum injection time was set to 60 msec. MS2 resolution was set to 15,000, quadrupole isolation 1.7 m/z, AGC of 1e5, dynamic exclusion of 45 sec and maximum injection time of 60 msec.

**Data processing**

Raw data was processed with MaxQuant v1.6.0.16. The data was searched with the Andromeda search engine against the SwissProt human proteome database (November 2018 version, 20397 entries), trypsin/P as the chosen protease, carbamidomethylation of C as a fixed modification and protein N-terminal acetylation and oxidation of M as variables. 2 miscleavages were allowed, precursor mass tolerance was set to 10 ppm, while the fragment ion was 20 ppm, the Q-value threshold was 0.01 and the maximal FDR was set to 0.01. Minimum peptide ratio was set to 1 and the LFQ quantification was performed using unique peptides only. Match between runs was enabled. The LFQ intensities were used for further calculation using Perseus v1.6.0.7. Decoy hits were filtered out, as well as proteins that were identified on the basis of a modified peptide only. The LFQ intensities were log transformed and only proteins that had at least 2 valid values in at least one experimental group were kept. The remaining missing values were imputed by a random low range distribution. Student’s t-tests were performed between the relevant groups to identify significant changes in protein levels.
